# Multiple endocrine neoplasia type 1 associated with breast cancer: A case report and review of the literature

**DOI:** 10.3892/ol.2014.2144

**Published:** 2014-05-13

**Authors:** YOUNG JU JEONG, HOON KYU OH, JIN GU BONG

**Affiliations:** 1 Department of Surgery, College of Medicine, Catholic University of Daegu, Nam-gu, Daegu 705-718, Republic of Korea; 2 Department of Pathology, College of Medicine, Catholic University of Daegu, Nam-gu, Daegu 705-718, Republic of Korea

**Keywords:** multiple endocrine neoplasia type 1 (MEN1), *MEN1* gene, breast cancer, pancreatic endocrine carcinoma, primary hyperparathyroidism

## Abstract

Multiple endocrine neoplasia type 1 (MEN1) is a cancer predisposition syndrome that includes a combination of endocrine and non-endocrine tumors. The present study reports a rare case of MEN1 associated with breast cancer with the *MEN1* gene mutation. A 45-year-old female was diagnosed with breast cancer subsequent to presenting with a right breast mass. Pre-operative radiological studies indicated right breast cancer with a suspicious metastatic nodule of the lung. Further studies demonstrated bilateral thyroid nodules, a neuroendocrine tumor of the pancreas, paraganglioma, a left adrenal adenoma, gallstones, uterine subserosal myoma and pituitary macroadenoma. Laboratory examinations revealed hypercalcemia, hypophosphatemia and an increased intact parathyroid hormone level. The workup for the suspected MEN syndrome revealed an increased basal plasma level of insulin-like growth factor-1, prolactin and calcitonin, and an increased 24-h urinary free cortisol level. The patient underwent surgical removal of the breast cancer and the tumors of the pancreas, adrenal gland, thyroid and parathyroid glands, uterus, anterior mediastinum and lung. The pathological diagnosis of the resected breast was of invasive ductal carcinoma. Otherwise the pathological diagnosis was of calcitonin-producing pancreatic endocrine carcinoma, adrenal cortical adenoma, bilateral papillary thyroid carcinomas, parathyroid adenomas, uterine leiomyoma with adenomyosis, a thymic carcinoid tumor and lung hamatoma. Gene analysis was performed to determine the association between gene mutations and the development of tumors in this patient, and a germ-line *MEN1* gene mutation was consequently detected. It could be assumed that MEN1 syndrome may have possibly predisposed the present patient to breast cancer. However, additional observations and further studies are required to demonstrate this association.

## Introduction

Multiple endocrine neoplasia type 1 (MEN1) is an autosomal dominant cancer predisposition syndrome (1), caused by mutations in the *MEN1* gene (2). The *MEN1* gene is located on chromosome 11q13 (2). Previous studies of loss of heterozygosity (LOH) by microsatellite analysis in tumor tissues of MEN1 patients have supported a tumor suppressor function of the *MEN1* gene (3–5). Although patients with MEN1 syndrome are characterized by the presence of tumors of the parathyroid gland, anterior pituitary and endocrine pancreas (6), it has been demonstrated that tumors may arise in over 20 different endocrine and non-endocrine organs in these patients. Less common manifestations in MEN1 patients include adrenocortical tumors, foregut carcinoid tumors, such as thymic carcinoid, bronchial carcinoid and gastric enterochromaffin-like tumors, and cutaneous/mucosal or visceral abnormalities, such as facial angiofibromas, lipomas, hypomelanotic macules, collagenomas and meningiomas (6,7). There is also a frequent association with thyroid tumors, however, this association should be considered likely causal for the high incidence of thyroid abnormalities in the general population (7).

The present study reports the case a patient with an unusual combination of MEN1-associated tumors and breast cancer. The patient exhibited major clinical manifestations of MEN1, such as primary hyperparathyroidism, pituitary adenoma and pancreatic endocrine carcinoma together with other tumors, including adrenocortical adenoma, a thymic carcinoid tumor, papillary thyroid carcinoma, uterine leiomyoma, lung hamatoma and breast cancer. Gene analysis was performed for the *MEN1*, *RET*, *BRCA1* and *BRCA2* genes to determine the association between gene mutations and the development of tumors in the patient. Written informed consent was obtained from the patient for publication of this case report and accompanying images.

## Case report

### Patient

A 45-year-old female presented to the Daegu Catholic University Hospital (Daegu, Korea) with a mass in the right breast that had been present for the previous two months. The patient had previously suffered no serious illnesses and had no known family history of malignancy, including breast cancer. The patients’s mother was known to have diabetes, but there was no known family history of MEN1.

### Physical examination and imaging

**Figure 1:**
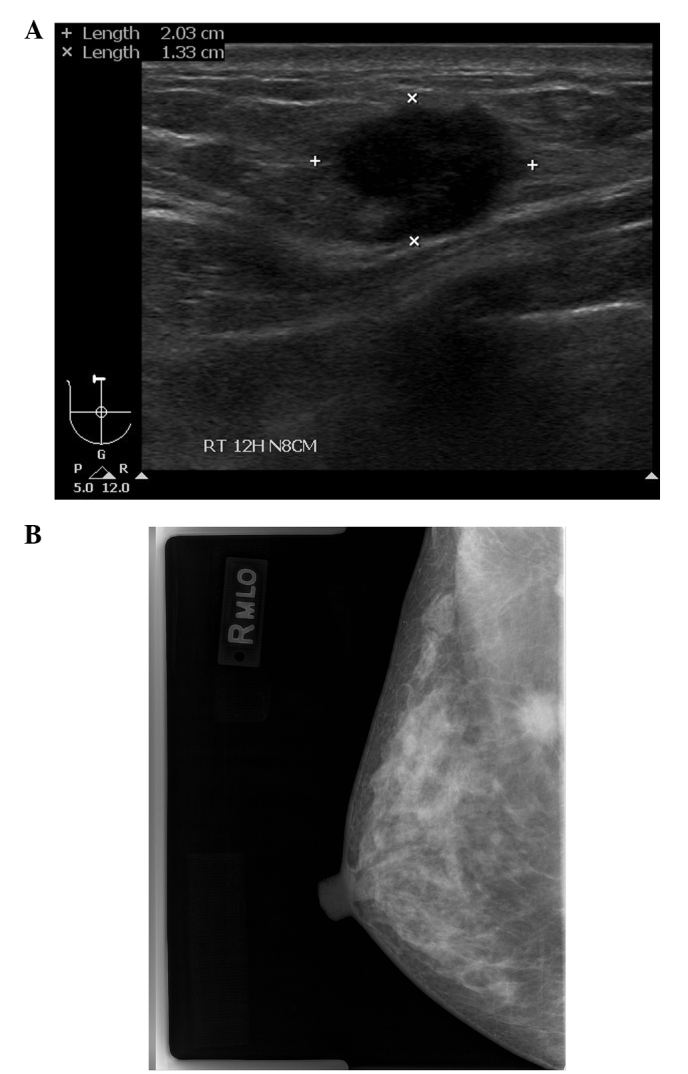
Radiological findings of the right breast. (A) Mammography showing a spiculate hyperdense lesion in the upper portion of the right breast. (B) Ultrasonography (USG) scan showing an irregularly-shaped hypoechoic lesion in the right breast.

**Figure 2:**
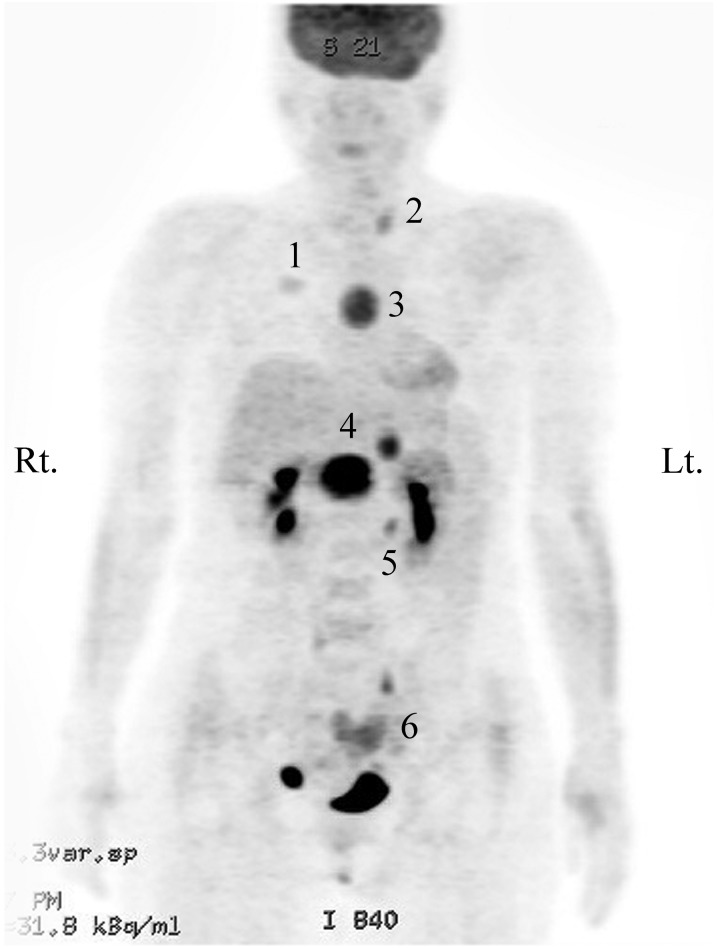
Findings of positron emission tomography-computed tomography (PET-CT). PET-CT showed (1) a metabolically mildly active right breast lesion compatible with the proven right breast cancer, which was proven by an ultrasound-guided core needle biopsy (2) a metabolically mildly active left thyroid lesion, which was suspicious for thyroid cancer, (3) a metabolically active prevascular (ascending paraaortic) nodal lesion, (4) metabolically active upper abdominal nodal lesions (left gastric lymph node and adjacent to hepatic and splenic artery), which were suspicious for metastatic nodal lesions or a primary pancreas tail mass (4), (5) suspicious left adrenal metastasis and (6) mild endometrial uptake, which was suspicious for myoma or endometrial malignancy.

Upon physical examination, a fixed, firm mass, 2 cm in diameter, was palpated without tenderness in the right breast. There was no clinical evidence of regional lymphadenopathy. Mammography revealed a spiculate hyperdense lesion in the upper portion of the right breast (Fig. 1A). Ultrasonography (USG) revealed an irregularly-shaped hypoechoic lesion in the right breast in accordance with the finding of the mammography (Fig. 1B). The patient underwent an ultrasound-guided core needle biopsy, which revealed the features of an invasive ductal carcinoma. Radiological studies, including computed tomography (CT) of the chest, magnetic resonance imaging (MRI) of the breast and positron emission tomography-CT (PET-CT) of the torso were conducted for pre-operative evaluation of the right breast cancer. PET-CT showed metabolically active lesions in the right breast, the anterior mediastinum, the peripancreatic area of the upper abdomen and the left adrenal gland, which corresponded to the lesions observed on the CT scan (Fig. 2). In addition, the left thyroid gland and the endometrium of the uterus showed mild FDG uptake on the PET-CT, and a suspicious metastatic nodule of the lung was observed on the CT scan. The findings of an additional abdominopelvic CT scan indicated a neuroendocrine tumor of the pancreas, paraganglioma, a left adrenal adenoma, gallstones and uterine subserosal myoma.

### Laboratory results

Concomitantly, laboratory examinations revealed hypercalcemia (11.8 mg/dl; normal range, 8.2–10.2 mg/dl), hypophosphatemia (2.0 mg/dl; normal range, 2.5–4.5 mg/dl) and an increased intact parathyroid hormone (iPTH) level of 340.8 pg/ml (normal range, 12–72 pg/ml). The workup for the suspected MEN syndrome revealed an increased basal plasma level of insulin-like growth factor-1 (430 ng/ml; normal range, 124–290 ng/ml), prolactin (43.9 ng/ml; normal range, 3–25 ng/ml) and calcitonin (286.3 pg/ml; normal range, <10 pg/ml), and an increased 24-h urinary free cortisol level (563.5 μg/24 h; normal range, 55.5–286.0). Basal plasma levels of other hormones, including growth hormone, thyroid stimulating hormone, adrenocorticotrophic hormone, gonadotrophic hormone, cortisol, aldosterone, plasma rennin activity, gastrin, insulin and urinary catecholamines, were all within normal limits.

### Imaging results

MRI brain scans showed a tumor of 1.4×0.9 cm in size at the posterior aspect of the adenohypophysis, which was indicative of a pituitary macroadenoma. USG of the neck revealed relatively well-defined hypoechoic nodules in the bilateral thyroid lobes. Fine-needle aspiration cytology for nodules at the inferior pole of the bilateral thyroid lobes showed a few atypical epithelial cells of suspected parathyroid origin.

### Treatment and outcome

**Figure 3:**
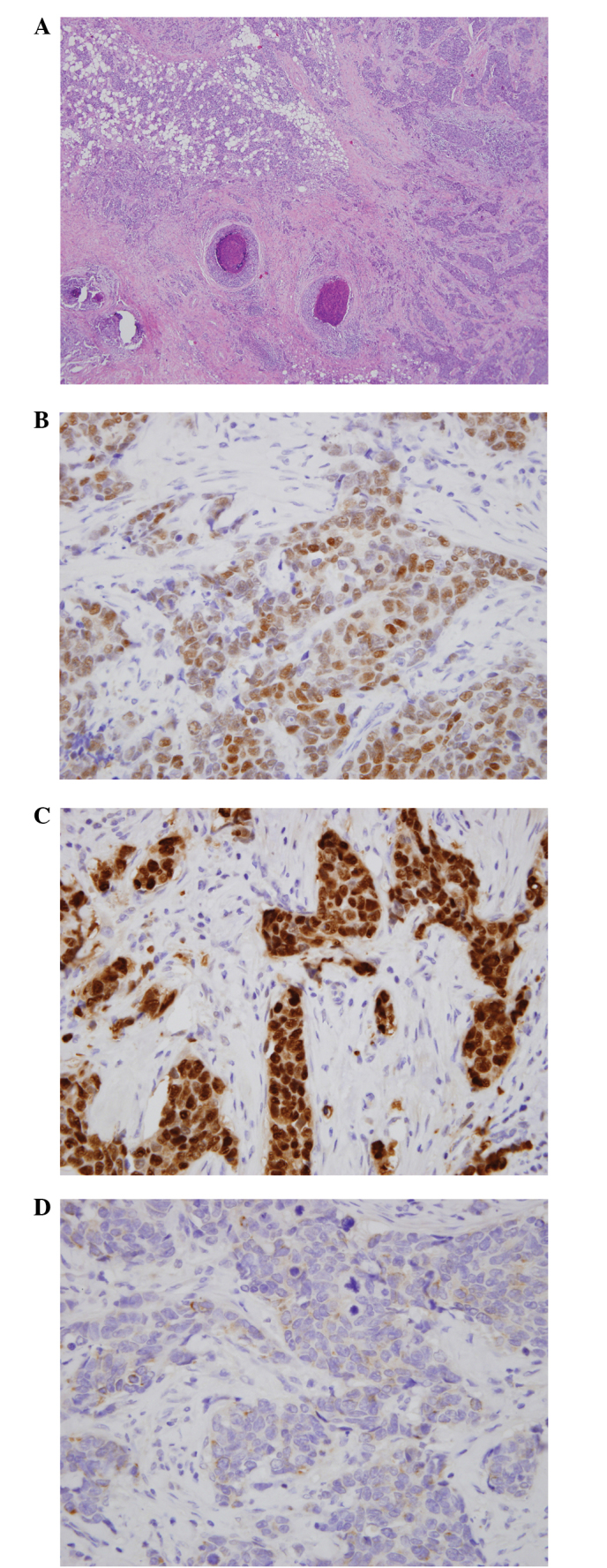
Histologic findings of the right breast mass after lumpectomy. (A) Microscopic findings of the specimen showing irregular infiltration of tumor cells, stromal fibrosis, periductal infiltration of tumor cells with intact basement membrane and calcified materials (HE stain; magnification, ×40). Immunohistochemically positive staining for (B) estrogen receptor (ER) and (C) progesterone receptor in the tumor (HE stain; magnification, ×400). (D) Imunohistochemical staining for HER2/neu proliferation showing negative findings (HE stain, ×400). HE, hematoxylin and eosin.

The patient underwent multiple pancreatic mass enucleation, left adrenalectomy, cholecystectomy and hysterectomy. The pathological diagnosis was of calcitonin-producing pancreatic endocrine carcinoma for the pancreatic mass, adrenal cortical adenoma for the adrenal mass, cholelithiasis and uterine leiomyoma with adenomyosis, respectively. A month later, a right breast lumpectomy with right axillary lymph node dissection, total thyroidectomy, parathyroidectomy, extended thymectomy and wedge resection of the lung were performed simultaneously. The pathological diagnosis of the resected breast was of an invasive ductal carcinoma associated with ductal carcinoma *in situ* demonstrating estrogen receptor (ER)-positive, progesterone receptor-positive and HER2/neu proliferation-negative breast cancer, and metastatic carcinoma was detected in the right axillary lymph nodes (Fig. 3). The pathological diagnosis of nodules in the thyroid gland, parathyroid gland, anterior mediastinal mass and lung nodule were bilateral papillary thyroid carcinomas, not medullary carcinoma, and parathyroid adenomas, a thymic carcinoid tumor and lung hamatoma, respectively. Subsequent to the surgery, the serum calcium levels and the iPTH decreased to within the normal range. The suspicious pituitary adenoma remained untreated and has not since changed in size in 2 years of follow-up examinations. Following the surgery, the patient received adjuvant chemotherapy with 4 cycles of Adriamycin and cyclophosphamide, followed by 4 cycles of docetaxel and then radiation therapy to the right chest and axilla. The patient is currently undergoing anti-estrogen therapy using tamoxifen, and has exhibited no evidence of local tumor recurrence or distant metastases in the 2 years since the surgery.

### Mutational analysis

Given the clinical impression of combined MEN1 and MEN2A based on the clinical manifestations of the patient, confirmatory genetic testing for the *MEN1,* as well as the *RET* gene was performed. Also, *BRCA1* and *BRCA2* genetic testing was performed to determine the association between gene mutations and the development of other tumors, including breast cancer.

Once informed consent had been obtained, peripheral blood samples were collected from the patient. Genomic DNA was extracted from blood using a commercial kit (Wizard Genomic DNA Purification kit; Promega, Madison, WI, USA). Polymerase chain reaction and mutational analyses of the genes were performed as previously described (8–10). All coding exons for the *MEN1* gene, and exons 10, 11, 13, 14, 15 and 16 of the *RET* proto-oncogene were analyzed by direct sequencing. The 22 exons and the exon-intron boundaries of the *BRCA1* gene and the 26 exons and the exon-intron boundaries of the *BRCA2* gene were analyzed by direct sequencing. DNA sequencing was performed on the pretreated PCR product using an automated direct sequence analyzer (ABI PRISM 3100 Genetic Analyzer; Applied Biosystems, Foster City, CA, USA).

### Results

The *MEN1* gene germline mutational analysis revealed a 5-bp duplication in exon 3, namely, c.196_200dupAGCCC, which resulted in a frameshift mutation of the *MEN1* gene. This mutation is one of the known germ-line mutations of the *MEN1* gene in MEN1 patients (11). In addition, a polymorphism of the *MEN1* gene was detected at codon 423 in exon 10 of the *MEN1* gene, with substitution of a cytidine to a thymidine (C423T), which did not cause a change of amino acid. Mutation analysis for the *RET*, *BRCA1* and *BRCA2* genes showed a polymorphism of the *RET* and *BRCA1* genes, but no significant mutation was detected in this patient.

## Discussion

The present study reports the case of a patient with MEN1-associated tumors and breast cancer, in which we identified germline mutations in *MEN1,* but not in *BRCA1/2*.

Although increasing evidence for MEN1-associated non-endocrine tumors has been reported, there are limited data on the association of breast cancer with MEN1. To the best of our knowledge, there have been two reports of MEN1 associated with breast cancer regardless of *BRCA1/2* germline mutations (12,13). Honda *et al* (12) reported a case with an unusual combination of primary hyperparathyroidism, primary aldosteronism and breast cancer, in a patient with a germline *MEN1* gene mutation, which is regarded as a benign polymorphism and loss of heterozygosity (LOH) of the *MEN1* locus in the DNA from breast cancer tissue. The study hypothesized that the clinical spectrum of MEN1 might include breast cancer. Recently, Inic *et al* (13) also reported the case of a patient with breast cancer and MEN1. Several other studies have also described cases of patients with MEN1 and a family history of breast cancer, however, in these studies, the breast cancer was caused by mutations of the *BRCA1/2* gene not the *MEN1* gene (14,15). Papi *et al* (14) reported the cases of carriers of both the *MEN1* and *BRCA1* germline mutations, who had a classical MEN1 phenotype with a family history of breast cancer. Ghataorhe *et al* (15) reported the case of a patient with both the *MEN1* and *BRCA2* germline mutations, who had MEN1 and a family history of male breast cancer.

The *MEN1* gene responsible for MEN1 acts as a tumor suppressor gene (16), and tumors in MEN1 arise through the two-hit mechanism (3). The first hit is a germline mutation, and the second hit is a somatic inactivation of the remaining wild-type allele in a single cell of certain tissues, which initiates neoplastic transformation (17). A wide variety of germline mutations of the *MEN1* gene have been identified to date (11,18). These observed mutations are scattered throughout the entire coding region and include nonsense, missense and frameshift mutations (11). In the present study the germline mutational analysis revealed a frameshift mutation in exon 3 of the *MEN1* gene, which is a known mutation of the *MEN1* gene associated with MEN1 syndrome (11,18). Several studies have indicated that mutation type or location within *MEN1* may be associated with clinical presentation (19,20). However, there is no apparent genotype-phenotype correlation (7,11). Although 196_200dupAGCCC, the *MEN1* germline mutation detected in the present study, has previously been reported in MEN1-related disorders (21–23), the clinical manifestations of the patient in the present study are different from those of previous studies, which indicates a lack of genotype-phenotype correlation.

The product of the *MEN1* gene, menin, is a nuclear protein whose interaction with several nuclear proteins indicates a role in transcriptional regulation (24–26). Previous studies support a role for *MEN1* in the control of cell growth and differentiation, and in sensing or repairing DNA damage (27–30). The loss of menin function in a tumor precursor cell is involved in the mechanism for tumor formation in MEN1 (1,20). In this regard, there are several possible mechanisms of involvement for *MEN1* in breast cancer formation. Menin has been proposed to be involved in signaling pathways that have a role in breast cancer formation, and it may also control cell cycle progression and genomic integrity (1). Honda *et a*l (12) hypothesized that an alteration of the *MEN1* gene with LOH and/or another tumor suppressor gene located in the *MEN1* locus on chromosome 11q13 may be involved in the development of breast cancer without somatic gene mutations. Data are conflicting as to how the loss of menin-ERα interaction is associated with breast carcinogenesis. Menin can directly interact with the ERα in a hormone-dependent manner (31). Also, menin has a demonstrable role as a coactivator for ERα-mediated transcription by increasing the methylation of lysine 4 of histone 3 and the consequent transcription of the trefoil factor-1 (*TFF1*) gene (26,31). The product of *TFF1* is estrogen-induced breast cancer-associated peptide, and this is indicated to be involved in breast carcinogenesis and a variety of other tumor progression mechanisms (31–35). Normal mammary tissue expresses little or no TFF1 protein expression in normal breast ducts (36,37), and TFF1 expression is increased and positively associated with ER-positive tumors in breast cancer (35). Several studies have shown that the protein expression of TFF1 is associated with an improved prognosis and inversely associated with histological grade (33,35).

In summary, the current study presented the rare case of a patient with MEN1 associated with breast cancer, in which a germline mutation of the *MEN1* gene was detected. In this patient, MEN1 syndrome may have predisposed the patient to developing breast cancer. However, there have been few studies regarding the association between breast cancer and MEN1 syndrome, and further studies and additional case reports are required to clarify this connection.
